# Ethical issues across different fields of forensic science

**DOI:** 10.1186/s41935-017-0010-1

**Published:** 2017-07-18

**Authors:** Praveen Kumar Yadav

**Affiliations:** Department of Forensic Science, Punjabi University, Patiala, 147002 India

**Keywords:** Ethics, Forensic science, Forensic psychology, DNA databases

## Abstract

Many commentators have acknowledged the fact that the usual courtroom maxim to “tell the truth, the whole truth, and nothing but the truth” is not so easy to apply in practicality. In any given situation, what does the whole truth include? In case, the whole truth includes all the possible alternatives for a given situation, what should a forensic expert witness do when an important question is not asked by the prosecutor? Does the obligation to tell the whole truth mean that all possible, all probable, all reasonably probable, all highly probable, or only the most probable alternatives must be given in response to a question? In this paper, an attempt has been made to review the various ethical issues in different fields of forensic science, forensic psychology, and forensic DNA databases. Some of the ethical issues are common to all fields whereas some are field specific. These ethical issues are mandatory for ensuring high levels of reliability and credibility of forensic scientists.

## Background

The difference between a trade and a profession is that the latter possesses a self-imposed code of conduct to which its members agree to submit. Such codes are usually ethical based and not imposed by outside legislations and hence members of each profession voluntarily adhere to such codes the members of a profession adhere to such codes. It is need-of-the-hour that each profession should create its set of codes that is a system of self-regulation. In absence of such self-regulatory codes, governments fill the gaps by imposing legal rules which are far less appropriate and suffer from serious shortcomings. The number of professionals working in the field of forensic science is relatively low in comparison to those working in other fields such as medicine and law. Since the interface between science and criminal justice system is filled by forensic scientists, therefore, many owe allegiance to other professional bodies. (Knight 1989; Murdock and Holmes 1991).

Forensic scientists help law enforcement officers, lawyers, judges, and juries in delivering justice by providing results and conclusion; hence they work in forensic science laboratories associated with law enforcement or other governmental agencies that have ethical codes developed specifically for their organizations. These codes address those areas of professional behavior that are important to the bulk of the people within that agency. Be it a police or prosecutor agency, ethical codes will be designed for police officers and prosecutors respectively. Since ethical codes are organization-specific and are applied partly to forensic scientists, then there is a need to establish codes that are specific to forensic scientists. Such codes will guide them in a situation where ethical issues arise from the type of work the criminalist does. What is ethical to one forensic scientist may be unethical to another and yet to another, therefore, in such situations; these codes may help to guide them to them to the most appropriate course of action. Moreover, such ethical codes stand for the hallmark of the professional status (Barnett 2001).

Many commentators have acknowledged the fact that the usual courtroom maxim that is “to tell the truth, the whole truth, and nothing but the truth” is not so easy to apply in practicality. In any given situation, what does the whole truth includes? In case the whole truth includes all the possible alternatives for a given situation, what should a forensic expert witness do when an important question is not asked by the prosecutor? Does the obligation to tell the whole truth mean that all possible, all probable, all reasonably probable, all highly probable, or only the most probable alternatives must be given in response to a question? (Barnett 2001).

Due to the absence of any regulatory organization, forensic science has no official recognition or registration that should provide certain rights and responsibilities to forensic scientists. In forensic science, individuals from various fields with adequate qualifications are employed without any statutory certification or registration. The only possible restraint upon professional misconduct of a forensic scientist is through membership of such a craft organization (Knight 1989). Therefore, there is an immense need to establish these codes, but this doesn’t mean that they can never be changed; on the contrary, they should be revised regularly from time to time to meet the growing demands of maintaining standards of forensic laboratories.

Laboratory results together with the expert’s opinion that interprets them must never be falsified, trimmed, tailored or otherwise modified to suit a third party, be the motive political, military, racial, financial or any other. Forensic scientists may work under threat of financial or career penalty or of physical violence to themselves or their families. Introducing a strong and widely publicized code of conduct helps to strengthen the resolve of the threatened scientist, especially if he knows that a strong body of internationally reputable colleagues are willing to expose any oppression or malpractice.

Improper conduct in the field of forensic science includes continued abuse of alcohol or drugs, criminal convictions for dishonesty or violence, defamation of professional colleagues etc. and is incompatible with those dealing with law enforcement on daily basis. In private, unfair practices to obtain clients, unacceptable means of advertising and false claims of expertise are present which must be discouraged.

## Professional Vs personal ethics

Forensic science is the interface of science and law where principles of science are used for legal purposes. Hence, the ethics of forensic science are the ethics pertaining to the application of science to law. Forensic science has many controversial ethical facets and forensic scientists are often surrounded by baffling ethical disputes. There persists an arbitrary distinction between ethics and morals which enables them to avoid many ethical dilemmas. Personal ethics or morals in the field of forensic science refer to ‘the concerns a forensic scientist has, that are based on personal ethics (morals) or religious considerations which are not derived from professional and/or scientific roles. On the contrary, the professional ethics refer to the codes or guidelines that regulate the professional and scientific conduct which are more fundamental compared to personal ethics or morals. (Weinstock et al. 2013).

## Ethical dilemmas in forensic science

Siegal (2012) has classified ethical dilemmas broadly into six categories.

### Professional credentials

These include misrepresentation of the credentials before the court of law. Misrepresentations include educational degree attainment (e.g. claiming an unearned Ph.D. or a degree was earned from a particular institution when, in fact, it was not), professional licensures or certifications (e.g. falsely claiming certification as a forensic Pathologist from the American Board of Pathology or a common tactic of equating having attained actual certification with being board “eligible”), employment history and data about previous testimonies such as number of times, locations, etc. Most often this is done to impress the client, the judge or jury to ward off the challenges such as cross-examination by exaggerating the qualifications. Due to lack of resources and fact-checking methods, such exaggerations are seldom caught, and such acts are unethical and must be dissuaded.

### Laboratory analytical procedures

Most laboratories have validated and well-established protocols to be followed during tests of analysis as well as recording these tests and their results. Laboratories place priority in the implementation of such protocols but, less than often, such protocols are not followed by the forensic scientist which is unethical. Unethical issues include making insufficient or indiscriminate analysis, and analysis to fit the written law. Sometimes forensic scientists report the results without even opening the containers; a practice known as ‘dry-labbing’. Results and conclusions offered by forensic scientists must be explicit and clear.

### Interpretation of analytical data and presentation of testimony in a court of law

In the courtroom, forensic scientists face many ethical dilemmas while providing their testimonies. Ethical dilemmas associated with the interpretation of analytical data and presentation of testimony in a court of law may include bias on the part of forensic scientists, use of scientific jargons, use of confusing or deceptive testimonies, excessive equivocacy, and advocacy. Another issue with forensic laboratories is the way results and conclusions are reported. Some laboratories report minimal results without any useful or appropriate explanations. Also, many a time, the forensic scientist who performed the analysis is not even required to be present in the court for the testimony.

### Privately employed forensic scientists

An increasing number of private consultant practices posed a serious likelihood to the ethics in the field of forensic science. No disciplinary code can be applied to such private consultants. The professional integrity of a member is perhaps less of a problem in forensic science than in other professions, as we possess or perhaps suffer the most stringent form of quality control in the form of cross-examination in courts, where any malpractice, omissions or fudging is very likely to be revealed. The private consultancy offers the greatest risk of malpractice as there are less supervision, less peer review, and more financial incentive.

### Publicly employed forensic scientists

In the case of public forensic laboratories, it must be noted that they are neither a part of nor administered by the local governments or local law enforcement agencies. This leads to the development of a usual perception that these laboratories are part of a local law enforcement agency. In such case, it is very necessary to maintain the autonomy of these laboratories to maintain a high ethical ground.

### Obligations to the forensic science profession and professional skill maintenance

As a scientist, researcher and practitioner, all forensic scientists have the innate responsibility and obligation towards the forensic science profession to maintain the higher ethical values and standards. Ethical dilemmas include three categories that are a failure to keep up to date with recent advancements and updated knowledge, improper use of proficiency tests, continuing the improper educational practice.

## Objectivity in forensic science

Applebaum (1997) pointed in his paper on ethics in forensic psychiatry that the basic principle of ethics is telling the truth and distinguishes between subjective and objective truth telling. Subjective truth telling is to state what we believe is true whereas the objective is to recognize the limitations of methods used to reach conclusions. It includes recognizing the limitations of our scientific and professional knowledge which has led to deduction of conclusions. It is objective to include literature in reports which support, as well as contradicts our conclusions in order to use the explanatory framework that is widely accepted by the scientific community. In his treatise on general psychotherapy, Jasper (1997) differentiated between the objective and subjective phenomena; he identified the objective as ay trained observer where they are perceived by our senses whereas the subjective cannot be perceived by sense organs. Norko (2005) stated that the subjective element of truth telling involves the expert’s honesty whereas the objective element relates to his knowledge and testimony.

Similarly, forensic scientists must remain objective while reaching conclusions that can be attained through training and following a standard ethical code. The ethical forensic scientist is a scientist who strives to reach conclusions based on examinations performed without any bias or extending themselves beyond their capabilities or talents (Murdock and Holmes 1991). They must not forget that objectivity is their main attribute and that they must examine all the angles before reaching conclusion. They have responsibilities towards the public, therefore, their examinations and analyses must be accountable and objective (Murdock and Holmes 1991).

## Ethical dilemmas in forensic psychology

Shapiro (2016) underlined the following ethical issues after reviewing various ethical codes present across the world.

### Misuse of work

All over the world, available ethical codes made attempts to make it unambiguous that the psychology profession must not be misused by psychologists and other organizations alike. Under no circumstances, the use of the profession must involve a deprivation of basic human rights (American Psychological Association 2010). In cases where any violation of ethical codes is discovered, one must try to either resolve the issue or minimize its effect. Psychologists need to avoid or refuse to participate in practices contrary to the legal, civil or moral rights of others as well as refuse to assist anyone who might use a psychologist’s knowledge to advise, train or supply information to anyone to violate human rights (Canadian Psychological Association 2000).

### Competence

It is stated that psychologists must work as per and to the best of their competence boundaries, based on their education, training, supervised experience, consultation, study or professional experience (American Psychological Association 2010). They either are or become reasonably familiar with the judicial or administrative rules governing the roles they play. To determine the competence, we must take into consideration the relative complexity and nature of the service to be provided, relevant training and experience, preparation and study they were able to devote to the matter, and the opportunity for consultation in a particular subject matter area (American Psychological Association 2010). It is the ethical duty of forensic psychologists to inform the referral source as to whether there is a known basis in either research or practice to answer the particular question being asked. They must avoid misrepresentation of research in any way. Awareness of legal and professional standards, law, rules and procedures involved must not lead to threatening or impairment of the rights of the affected individual as well as being sensitive to and knowledgeable about individuals (Canadian Psychological Association 2000).

### The basis for scientific and professional judgments

These must be based on established scientific and professional knowledge, up to date research, with relevant literature and continuing education (American Psychological Association 2010; Canadian Psychological Association 2000).

### Delegating work to others

Legally known as Vicarious Liability, is the concept of supervision, where the supervisor is responsible for the work of those under his supervision. He must take reasonable steps to avoid delegating the work to people who have some sort of multiple relationships with those being served, that would lead to exploitation or lack of objectivity. (American Psychological Association 2010; Canadian Psychological Association 2000).

### Avoiding harm

Although it is rare for a psychologist to use this professional excellence to harm someone deliberately, sometimes the situation arises where harm is delivered unintentionally. Forensic psychologists must consider long-term harms before giving any evaluation. One such example of unintentional harm is a case where a forensic psychologist has to review execution of a criminal. If the culprit is found competent to be executed, he then will be causing harm to a life. In a counter argument, many will believe that if the culprit is not found competent to be executed, he is saving a life as well (Shapiro 2016).

### Multiple relationships

It is defined as being in a professional role with a person and at the same time in another with the same person or a closely associated person or promising to enter into another relationship with a person or a closely related person (Shapiro 2016). If the psychologist is required by law, institutional policy or unusual circumstances, to serve multiple roles in legal proceedings, they must clarify their role expectations and the extent of confidentiality at the outset and as circumstances change (American Psychological Association 2010). In addition, forensic psychologists must not assume a professional role if they have any other interest that could possibly impair their competency, objectivity or effectiveness in doing psychological work (American Psychological Association 2010).

Shapiro (2016) presented a case that illustrated the dilemma of multiple relationships in a vivid manner. The psychologist in question was ordered by the court to provide treatment to a child who was sexually abused and to provide periodic reports to the court regarding the same case. She, at all times, regarded her role as a therapist, not a forensic examiner. Later, a complaint was filed by the alleged abuser regarding the incompetency of the psychologist on the grounds that she didn’t follow the ethical guidelines. The psychologist stated that her role, was not forensic, it was therapeutic, and for that reason, she did not need to follow the guidelines. However, she had not made this issue clear until the complaint was filed. In other words, at the time she accepted the child under her supervision, she failed to make herself clear that she was only providing the therapeutic services and that her reports were not to be considered for forensic evaluation.

### Exploitation

A forensic psychologist must not exploit those whom one supervises or in which an authority role exists, such as clients, patients, students, supervisees, research participants and employees to further political or business interests, or the best interests of the research participants, students or employees. This exploitation may include soliciting of clients, sexual relationships or frightening individuals into receiving services.

### Informed consent

One must seek the consent of both the person involved and the representing council. An attorney must be obtained if the person is legally incapable of providing the consent himself. The forensic psychologist must inform the individual about the different parameters related to the anticipated services such as the limits of confidentiality. Oral consent can be taken in cases where the written consent cannot be obtained. However, in this case, the intervention of examination must be clearly stated and explained to the individual (Shapiro 2016).

There is an intense obligation on the part of the psychologist to find out whether the client is represented by a council or not. The fees, previous personal or professional relationships or any such parameters which can affect the relationship in later stages must be sorted out at the beginning (American Psychological Association 2013). The forensic psychologist must take into consideration what might cause a possible bias such as conflict of interest, examiner’s competence, and the scientific basis or limitations. The informed consent also extends to collateral sources of information that might affect their decision to participate (American Psychological Association 2010).

### Confidentiality

It is the prime obligation of a forensic psychologist to take reasonable precautions to protect the client’s confidentiality and must make it clear as or its limits. The disclosure must be made only with the client’s consent or consent of the legally authorized individual; it can also be made without the client’s consent only if mandated by law or when the psychologist uses the information for consultation or protection of the client (American Psychological Association 2010).

### Forensic methodology

It is the duty of the forensic practitioner that he must not withhold, distort or modify any relevant information, misinterpret the available evidence, and attempt to avoid or deny the contrary evidence. The forensic psychologist must not make any premature conclusions. Once the conclusion is reached, the psychologist must advocate his opinions forcefully and with appropriate vigor (American Psychological Association 2013). He must accurately represent his credentials, avoiding misrepresentation and maintaining competence in areas of practice and specialty (Canadian Psychological Association 2000). Information reporting must be as accurate not to lead to any alternative hypothesis (Meta code of Ethics 2005).

### Documentation

Proper records must be maintained to facilitate the provision of research, institutional requirements, accuracy in billing and compliance with the law. Obviously, confidentiality must still be maintained even while maintaining their records. However, these records can be used in emergency circumstances when required (American Psychological Association 2010). Records must be enough to support the continuity and appropriate coordination of activities with those of others (Canadian Psychological Association 2000).

### Assessment

It must be based on sufficient data including a personal examination unless it is not practical. Tests used for the assessment must be reliable and validated. Also, the strengths and limitations of the test data must be discussed.

## Ethical dilemmas in forensic genetics

Wallace (2014) and Cordner (2001) outlined the ethical issues related to the forensic database.

### Collection and storage of DNA samples

Obtaining samples for DNA database is one of the biggest and most debated upon issues. The first issue is who will be providing samples in respect to the criminal investigation? The second issue is who will be required to provide a sample, the profile from which will be stored in DNA database (Cordner 2001)?

The UK National DNA database was first forensic DNA database established in 1995. Its expansion to include DNA profiles of millions of innocent citizens into the database was widely criticized for it was considered as a breach of personal space. In 2008, the Grand Chamber of the European Court of Human Rights in the case of Marper vs. the UK reached a unanimous judgment that the indefinite retention of innocent people’s DNA profiles, fingerprints and samples breached privacy (Wallace et al. 2014). Different countries have different criteria to whose DNA sample must be obtained. For instance, in Australia, samples of those who are suspected of having committed crimes for which the penalty is more than 5 years are taken. Samples and profile must be destroyed within 12 months if the charge sheets are not filed, the prosecution is abandoned, or the accused is acquitted. On the other hand, if the accused has been convicted, his profile is retained in the database. In the UK, the profiles of all the accused of recordable crimes that include offenses for which there is a jail term if convicted, are recorded in the database. In France, the database is reserved only for those accused of sexual crimes (Cordner 2001). An audit system to monitor and prevent the unethical use of such databases is also vital.

### Testing the samples/ using the results

Correct sample collection, security, transport, storage along with processing and analysis are important conditions required for the high-quality database management. Meeting these conditions results in high level of confidence which in turn results in high reliability and high credibility. Contamination problems are a severe predicament and strong reason for doubtful credibility. Contamination can occur at various steps of analytical procedures such as sample collection, securing, transport, storage, and analysis. Although contaminations can never be prevented, it is imperative that they must be minimized and estimation errors are calculated (Cordner 2001).

### Access and retention of DNA samples

DNA profiles are stored in databases. Many authors claim the correlation of DNA markers with the physical characteristics. Moreover, DNA might reveal illness history. In pursuit of establishing such correlations and investigating them, researcher tends to overuse these databases. This will further put pressure on other databases to provide information for researcher’s assistance (Cordner 2001).

### Misuse of genetic research in application of genetics to forensic sciences

One of the discussed topics relate to the ethnic and racial labels to the genetic samples. Often forensic scientists try to use results from genetic research to put ethnic and racial labels on the samples encountered on the crime scene. However, many authors believe that the ethnic and racial differences are cultural in nature rather than biological or genetic. Many have raised questions on the scientific utility of racial classifications.

## Conclusion

Scientists and researchers are always baffled by the role of ethics. In multiple situations, ethics and research cross the path. Ethics are the soul of any profession and without it, the meaning of profession is vague and ambiguous. Ethics also help in establishing quality, validity, and authenticity of the profession. Although what is ethical to one person may be unethical to another, principles of ethics must be followed. Forensic science deals with the legal aspects and may help in establishing the guilt or exonerating the accused. Therefore, it is mandatory for every forensic organization to have an ethical code which guides forensic scientists to perform their duty with honesty and passion. Definition and limits of following ethical guidelines may vary from person to person but the minimum set of ethics must be made mandatory to be followed especially in the field of forensic science.
